# Impact of Type of Postoperative Complications on Long-Term Survival of Gastric Cancer Patients: Results From a High-Volume Institution in China

**DOI:** 10.3389/fonc.2021.587309

**Published:** 2021-10-11

**Authors:** Hua-Yang Pang, Lin-Yong Zhao, Hui Wang, Xiao-Long Chen, Kai Liu, Wei-Han Zhang, Kun Yang, Xin-Zu Chen, Jian-Kun Hu

**Affiliations:** Department of Gastrointestinal Surgery and Laboratory of Gastric Cancer, State Key Laboratory of Biotherapy, West China Hospital, Sichuan University and Collaborative Innovation Center for Biotherapy, Chengdu, China

**Keywords:** gastric cancer, severe complications, infectious complications, non-infectious complications, overall survival

## Abstract

**Background:**

This study aimed to evaluate the impact of postoperative complication and its etiology on long-term survival for gastric cancer (GC) patients with curative resection.

**Methods:**

From January 2009 to December 2014, a total of 1,667 GC patients who had undergone curative gastrectomy were analyzed. Patients with severe complications (SCs) (Clavien–Dindo grade III or higher complications or those causing a hospital stay of 15 days or longer) were separated into a “complication group.” Univariate and multivariate analyses were performed to reveal the relationship between postoperative complications and long-term survival. A 2:1 propensity score matching (PSM) was used to balance baseline parameters between the two groups.

**Results:**

SCs were diagnosed in 168 (10.08%) patients, including different etiology: infectious complications (ICs) in 111 (6.66%) and non-infectious complications (NICs) in 71 (4.26%) patients. Multivariate analysis showed that presence of SCs (P=0.001) was an independent prognostic factor for overall survival, and further analysis by complication type demonstrated that the deteriorated overall survival was mainly caused by ICs (P=0.004) rather than NICs (P=0.068). After PSM, patients with SCs (p=0.002) still had a significantly decreased overall survival, and the presence of ICs (P=0.002) rather than NICs (P=0.067) showed a negative impact on long-term survival.

**Conclusion:**

Serious complications, particularly of an infectious type, may have a negative impact on overall survival of GC patients. However, additional multicenter prospective studies with larger sample size are required to verify this issue.

## Introduction

Gastric cancer (GC) is a common malignancy in the world (1). Supported by advances in resection techniques and adjuvant therapies, surgical therapy has been the primary treatment for GC, which provides the opportunity to dramatically extend the long-term survival of GC patients (2–4). However, surgery for GC remains technically demanding, and the following postoperative complications have been reported to occur with a wide range of incidence: 7–46% (5–8).

Recent studies have shown that postoperative complications increase the length of hospital stay and early mortality (9, 10). Postoperative complications also decrease the overall survival (OS) and disease-free survival (DFS) in several types of cancer like lung, breast, and colon (11–13). In the contemporary field of gastric cancer research, the impact of postoperative complications on long-term survival of GC patients has also been suggested (14). Decreased OS and DFS in GC patients have been reported in retrospective series (15–19) and a recent published meta-analysis (20). Nevertheless, most of these studies did not exclude patients who died in the immediate postoperative period when assessing long-term survival. Of note, any potential impact of postoperative complications on cancer progression will be overshadowed by short-term increased mortality (21, 22). Moreover, few studies (19, 23) have yet discussed which specific type of postoperative complications (infectious and non-infectious complications) in GC patients most significantly impacts the patient’s long-term chances of survival.

The aim of this study was to explore the relationship between postoperative complication and its etiology and long-term survival.

## Methods

### Patients and Ethical Issues

**Figure 1:**
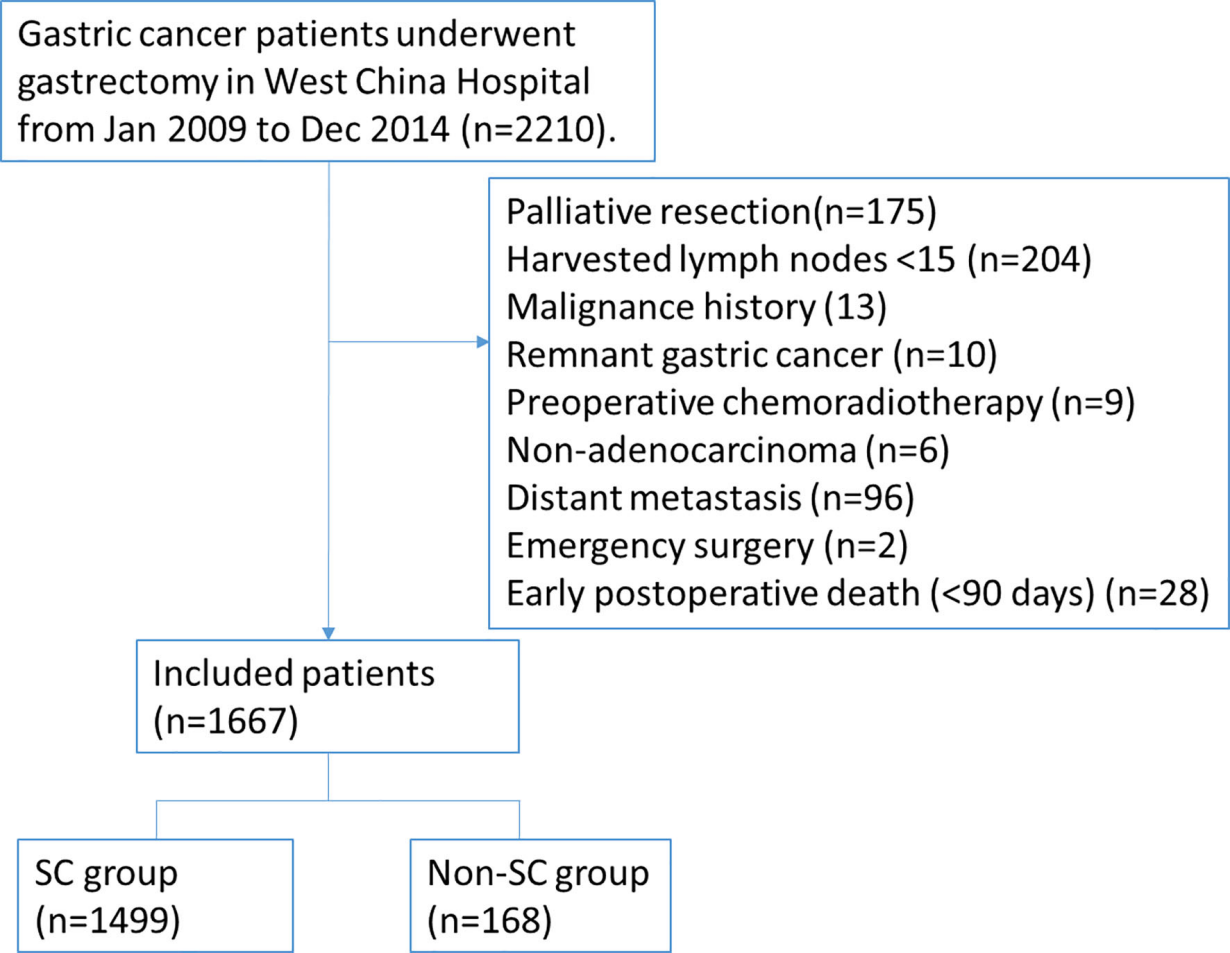
The flow diagram of gastric cancer patients enrolled in this study.

A total of 2,210 consecutive patients with gastric cancer who had previously undergone a gastrectomy procedure were selected from the database of Surgical Gastric Cancer Patient Registry in West China Hospital (WCH-SGCPR) from January 2009 to December 2014, with registration number WCH-SGCPR 2018-03. The present study involving human participants were reviewed and approved by the Research Ethics Committee of West China Hospital. Written informed consent from the patients/participants’ legal guardian/next of kin was not required to participate in this study in accordance with the national legislation and the institutional requirements. The inclusion criteria were as follows: (1) histologically proven gastric adenocarcinomas; (2) with radical surgical resection (R0); (3) without preoperative therapy; (4) no distant metastasis. The exclusion criteria of our study were patients with: (1) other synchronous or metachronous (within 5 years) cancers; (2) remnant gastric cancer; (3) harvested number of lymph node less than 15; (4) emergency treatment. Additionally, patients who died within 90 days (all the deaths were directly associated with serious intra- or postoperative complications) of the surgery were excluded to avoid exaggerating the effect of complications on long-term survival. Finally, 1,667 patients who underwent gastrectomy with potentially curative resection were included in this study, as shown in **Figure 1**.

Potentially curative resection is regarded as gastrectomy with no gross residual disease, combined with adequate lymphadenectomy. The surgery was performed by experienced surgeons and followed the Japanese gastric cancer treatment guidelines (24). The resected specimens were pathologically classified according to JGCA classifications (25) and staged with the updated AJCC 8th TNM system (26).

### Assessment of Postoperative Complications

In the present study, the Common Terminology Criteria for Adverse Events (CTCAE) version 4.0 (27), which is exhaustive enough in terms of postoperative morbidities, was used to define complications. As described by Song et al., we defined the severe complication (SC) group as patients with Clavien–Dindo (CD) grade III or higher complication or any morbidity causing a hospital stay of 15 days or longer (28–30). If a patient suffered from more than one complication, the highest-ranked complication was used for grade analysis.

All complications were categorized as infectious complications (ICs) or non-infectious complications (NICs). ICs included pulmonary infection, abdominal abscess (excluding leakage-related abscess), anastomotic leakage, wound infection, pancreatic leakage, pancreatitis, intestinal leakage, cholecystitis, urinary system infection, appendicitis, and bacteremia. NICs included gastroparesis, intestinal obstruction, intra-abdominal hemorrhage, pleural effusion, ascites, atelectasis, delirium tremens, respiratory failure, heart failure, arrhythmia, deep venous thrombosis.

### Follow-Up

The follow-up was mainly performed through outpatient visits. All patients were recommended to undergo follow-up every 3 to 6 months during the first 3 years and at least once yearly during the subsequent years. Follow-up information was also collected from the database and updated to January 1, 2020. In the 1,667 patients, 49 of them lost contact during the follow-up process; the response rate was 97.06% with the median follow-up time 89.50 (range, 3.00–129.80) months. The main reasons for failed follow-ups were changes of telephone number and address, or the patient’s refusal to attend to outpatient interview of our hospital.

### Statistical Analysis

For comparisons between patients with and without SCs, the Mann-Whitney U test was used to compare ordinal variables, whereas the Chi-square test or the Fisher exact test was used for unordered categorial variables. Then, multivariate logistic regression analysis was performed to detect independent risk factors for SCs. The Kaplan–Meier method and the log-rank test were used to calculate survival rates and compare survival rates, respectively. Univariate and multivariate Cox proportional hazards regression models were used to analyze the hazard ratios for a patient’s overall survival. Factors with a P value <0.1 in the univariate analysis as well as those that were clinically significant were entered into the multivariate model using an “Enter” method (31). A P value less than 0.05 (two-sided) was considered to be statistically significant.

To balance the potential confounders between the two groups, a 2:1 propensity score matching (PSM) was performed with the following variables: age, sex, comorbidities, extent of lymphadenectomy, perioperative blood transfusion, tumor size, tumor location, tumor invasion depth, and nodal involvement. A 0.2-width caliper of the standard deviation of the logit and the nearest neighbor matching was used to match across the two groups (32). All of these statistical analyses were performed using SPSS software version 23.0 (IBM, Armonk, NY, USA) and R version 3.6.0.

Considering the retrospective nature of this study, we would calculate the statistical power *via* PASS 11 (version 11.0.7).

## Results

### Description of Enrolled Study Population Cohort

The details of postoperative complications and characteristics of the included 1,667 patients with gastric cancer are presented in **Table S1** and **Table 1**. Postoperative complications occurred in 675 (40.49%) out of 1,667 patients, including 631 (37.85%) patients with CD grade I/II and 44 (2.64%) patients with CD grade III/IV complications. The non-SC group consisted of 992 (59.51%) patients without complications and 507 (30.41%) patients with complications less than 15 days’ hospital stay. The SC group consisted of 44 (2.64%) patients with CD grade III or higher complications and 124 (7.44%) patients with CD grade I/II complications causing a hospital stay of 15 days or longer. Further, in the SC group, 111 (6.66%) patients were found to have severe ICs and 71 (4.26%) patients to have severe NICs.

**Table 1:** Baseline characteristics of gastric cancer patients before and after propensity score matching.

	Primary cohort (n = 1,667)			PSM cohort (n = 503)		
	Non-SC group (n = 1,499)	SC group (n = 168)	P value	Non-SC group (n = 335)	SC group (n = 168)	P value
Age, year			<0.001			0.928
<65	1,126 (75.1)	102 (60.7)		202 (60.3)	102 (60.7)	
≥65	373 (24.9)	66 (39.3)		133 (39.7)	66 (39.3)	
Gender			0.059			0.986
Male	1,027 (68.5)	127 (75.6)		253 (75.5)	127 (75.6)	
Female	472 (31.5)	41 (24.4)		82 (24.5)	41 (24.4)	
Preoperative albumin, g/L			0.971			0.552
<35	162 (10.8)	18 (10.7)		42 (12.5)	18 (10.7)	
≥35	1,337 (89.2)	150 (89.3)		293 (87.5)	150 (89.3)	
Comorbidities			0.015			0.756
No	1,113 (75.3)	110 (65.5)		224 (66.9)	110 (65.5)	
Yes	386 (24.7)	58 (34.5)		111 (33.1)	58 (34.5)	
Surgery approach			0.807			0.914
Open	1,330 (88.7)	148 (88.1)		294 (87.8)	148 (88.1)	
Laparoscopic	169 (11.3)	20 (11.9)		41 (12.2)	20 (11.9)	
Gastrectomy			0.723			0.569
Partial	1,090 (72.7)	120 (71.4)		231 (69.0)	120 (71.4)	
Total	409 (27.3)	48 (28.6)		104 (31.0)	48 (28.6)	
Lymphadenectomy			0.058			1.000
<D2	48 (3.2)	1 (0.6)		2 (0.6)	1 (0.6)	
≥D2	1,451 (96.8)	167 (99.4)		333 (99.4)	167 (99.4)	
Resection of other organs			0.352			0.262
No	1,441 (96.1)	159 (94.6)		324 (96.7)	159 (94.6)	
Yes	58 (3.9)	9 (5.4)		11 (3.3)	9 (5.4)	
Perioperative blood transfusion			0.055			0.430
No	1,272 (84.9)	133 (79.2)		275 (82.1)	133 (79.2)	
Yes	227 (15.1)	35 (20.8)		60 (17.9)	35 (20.8)	
Tumor size, cm			0.525			0.539
<5	681 (45.4)	72 (42.9)		134 (40.0)	72 (42.9)	
≥5	818 (54.6)	96 (57.1)		201 (60.0)	96 (57.1)	
Tumor location			0.756			0.992
U/M/L	1,367 (91.2)	152 (90.5)		303 (90.4)	152 (90.5)	
Multiple	132 (8.8)	16 (9.5)		32 (9.6)	16 (9.5)	
Macroscopic type			0.546			0.259
Bormann 0–2	946 (63.1)	110 (65.5)		202 (60.3)	110 (65.5)	
Bormann 3–4	553 (36.9)	58 (34.5)		133 (39.7)	58 (34.5)	
Histological differentiation			0.308			0.807
G1/G2	460 (30.7)	58 (34.5)		122 (33.4)	58 (34.5)	
G3/G4	1,039 (69.3)	110 (65.5)		223 (66.6)	110 (65.5)	
Depth of invasion			0.486			0.636
T1/2/3	783 (52.2)	83 (49.4)		173 (51.6)	83 (49.4)	
T4	716 (47.8)	85 (50.6)		162 (48.4)	85 (50.6)	
Nodal involvement			0.474			0.859
N0	486 (31.2)	57 (33.9)		111 (33.1)	57 (33.9)	
N+	1,031 (68.8)	111 (66.1)		224 (66.9)	111 (66.1)	
Pathological stage			0.495			0.933
I	367 (24.5)	35 (20.8)		74 (22.1)	35 (20.8)	
II	340 (22.7)	43 (25.6)		82 (24.5)	43 (25.6)	
III	792 (52.8)	90 (53.6)		179 (53.4)	90 (53.6)	
Adjuvant chemotherapy			0.375			0.926
No	740 (49.4)	89 (53.0)		176 (52.5)	89 (53.0)	
Yes	759 (50.6)	79 (47.0)		159 (47.5)	79 (47.0)	

Compared with patients in the non-SC group, SC group had an older age (P<0.001) and more preoperative comorbidities (P=0.015), and tended to have higher proportion of males (P=0.059), higher frequency of D2/D2+ lymph node dissection (P=0.058) and preoperative blood transfusion (P=0.055). After PSM, the baseline characteristics became comparable between the two groups (all P values >0.05).

### Predictors Related to Occurrence of SCs

Relationships between the occurrence of SCs and clinicopathological parameters are shown in **Table 2**. Multivariate analysis identified that age 65 or higher (OR=1.815; 95%CI: 1.290–2.555; P=0.001) was the only independent risk factor for the development of SCs.

**Table 2:** Multivariable logistic regression analysis of risk factors for severe postoperative complications.

	Multivariable analysis	
	OR (95% CI)	P value
Age, year		
≥65 *vs.* <65	1.815 (1.290–2.555)	0.001
Sex		
Female *vs.* Male	0.721 (0.497–1.045)	0.084
Comorbidities		
Yes *vs.* No	1.262 (0.874–1.824)	0.214
Lymphadenectomy		
≥D2 *vs.* <D2	6.593 (0.898–48.404)	0.064
Perioperative blood transfusion		
Yes *vs.* No	1.274 (0.831–1.953)	0.266

### Prognostic Significance of SCs on Long-Term Survival

**Figure 2:**
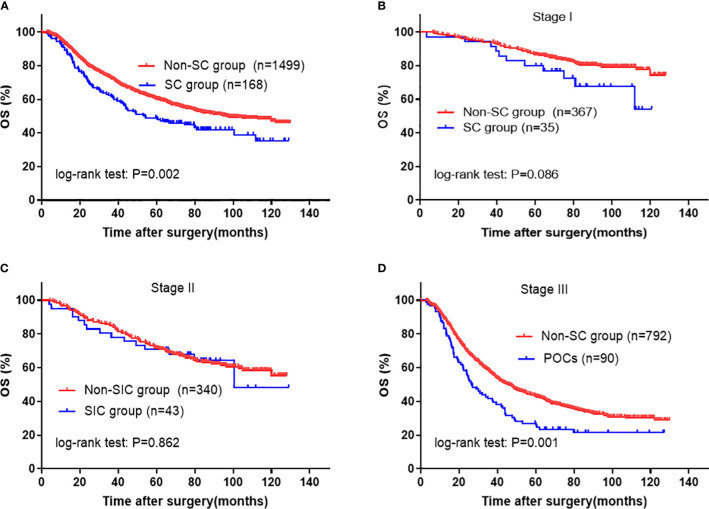
Kaplan-Meier survival analysis of SC in entire cohort. **(A)** in all patients; **(B)** in Stage I, **(C)** in Stage II, and **(D)** in Stage III patients. The significance of the difference between survival curves was calculated by the log-rank test. SC, severe complication.

As shown in **Figure 2A**, patients with SCs had a significant worse OS compared to those without (5-year OS rate 48.5 *vs.* 60.1%; P=0.002). The overall survival curves stratified by pathological stages are shown in **Figures 2B–D**. The curves were significantly separated in stage III cancers with P=0.001; however, no statistically significant difference was observed between stages I and II. Multivariable Cox regression analysis revealed that SCs (HR=1.442; 95% CI: 1.160–1.791; P=0.001) along with age, tumor size, histological grade, tumor invasion depth, nodal involvement, and adjuvant chemotherapy were independent prognostic factors for OS (**Table 3**). The statistical power of SCs on OS was 0.999 in this sample size.

**Table 3:** Univariate and multivariate COX regression analysis of prognostic factors for overall survival in primary cohort.

Variables	No. of patients	Univariate P value	Multivariate analysis*		Multivariate analysis^#^	
			HR (95% CI)	P value	HR (95% CI)	P value
Age (≥65 *vs.* <65)	439/1,228	<0.001	1.232 (1.049–1.446)	0.011	1.232 (1.049–1.446)	0.011
Gender (Male *vs.* female)	1,154/513	0.387				
Preoperative albumin (≥35 *vs.* <35 g/L)	1,487/180	0.600				
Comorbidities (Yes *vs.* No)	444/1,223	0.076	0.991 (0.839–1.173)	0.920	0.992 (0.838–1.173)	0.921
Surgery approach (Laparoscopic *vs.* Open)	189/1,478	0.001	0.829 (0.645–1.065)	0.143	0.830 (0.646–1.066)	0.144
Gastrectomy (Total *vs.* Partial)	457/1,210	<0.001	1.160 (0.993–1.355)	0.061	1.159 (0.992–1.354)	0.063
Lymphadenectomy (≥D2 *vs.* <D2)	1,618/46	0.692				
Resection of other organs (Yes *vs.* No)	67/1,600	0.434				
Perioperative blood transfusion (Yes *vs.* No)	262/1,405	<0.001	1.192 (0.987–1.428)	0.067	1.199 (0.993–1.447)	0.059
Tumor size (≥5 *vs.* <5 cm)	914/753	<0.001	1.436 (1.209–1.706)	<0.001	1.434 (1.207–1.703)	<0.001
Macroscopic type (Bormann 3–4 *vs.* 0–2)	611/1,056	<0.001	1.017 (0.870–1.189)	0.833	1.015 (0.868–1.186)	0.854
Histological grade (G3/G4 *vs.* G1/G2)	1,149/518	<0.001	1.192 (1.011–1.406)	0.037	1.190 (1.009–1.403)	0.039
Depth of invasion (T4 *vs.* T1/2/3)	801/866	<0.001	1.903 (1.609–2.252)	<0.001	1.903 (1.609–2.252)	<0.001
Nodal involvement (N+ *vs.* N0)	1,142/525	<0.001	2.239 (1.839–2.726)	<0.001	2.247 (1.845–2.736)	<0.001
Adjuvant chemotherapy (Yes *vs.* No)	838/829	0.918	0.806 (0.698–0.931)	0.003	0.808 (0.699–0.933)	0.004
**SCs (Yes *vs.* No)**	**168/1,449**	**0.002**	**1.442 (1.160–1.791)**	**0.001**		
**ICs (Yes *vs.* No)**	**111/1,556**	**0.003**			**1.455 (1.125–1.881)**	**0.004**
**NICs (Yes *vs.* No)**	**71/1,596**	**0.072**			**1.355 (0.977–1.878)**	**0.068**

*Multivariate analysis describing the prognosis of SCs for gastric cancer patients.

^#^Multivariate analysis describing the prognosis of ICs and NICs for gastric cancer patients.

The bold values indicate the main objects of this study.

**Figure 3:**
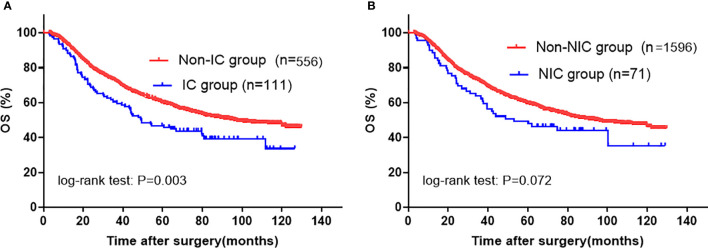
Kaplan-Meier survival analysis according to specific SC in entire cohort. **(A)** IC; **(B)** NIC. The significance of the difference between survival curves was calculated by the log-rank test. SC, severe complication; IC, infectious complication; NIC, non-infectious complication.

To clarify which type of complication had a contribution on poor OS, we performed univariate and multivariate analyses using each complication (ICs and NICs) with other parameters. In the univariate analysis, patients with ICs (5-year OS rate 45.9 *vs.* 59.8%; P=0.002) showed a decreased OS when compared with those without ICs, and patients with NICs (5-year OS rate 47.8 *vs.* 59.5%; P=0.072) also tended to have a worse prognosis when compared with those without NICs **(****Figures 3A, B**; **Table 3****)**. Further, multivariate analysis demonstrated that only ICs (HR, 1.455; 95% CI: 1.125–1.881; P=0.004) rather than NICs (HR, 1.355; 95% CI: 0.977–1.878; P=0.068) were independent risk factors for unfavorable OS **(****Table 3****)**. The statistical power values of ICs and NICs on OS were 0.997 and 0.925, respectively.

### Propensity Score Analysis

**Figure 4:**
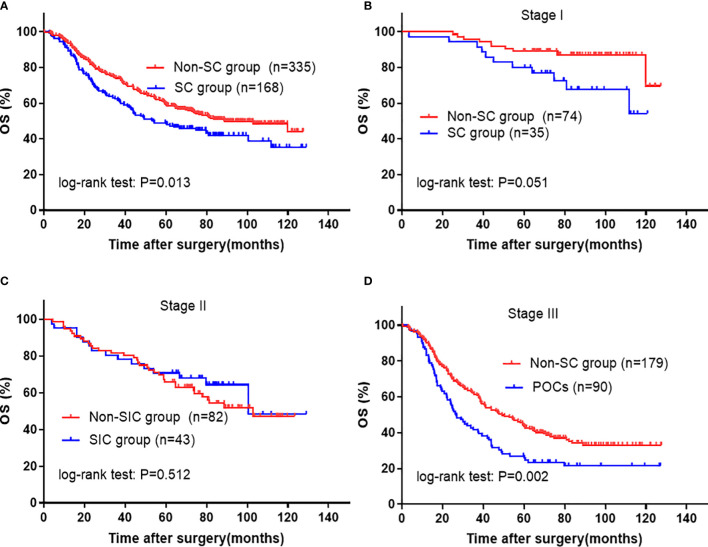
Kaplan-Meier survival analysis of SC in PSM cohort. **(A)** in all patients; **(B)** in Stage I, **(C)** in Stage II, and **(D)** in Stage III patients. The significance of the difference between survival curves was calculated by the log-rank test. SC, severe complication.

**Figure 5:**
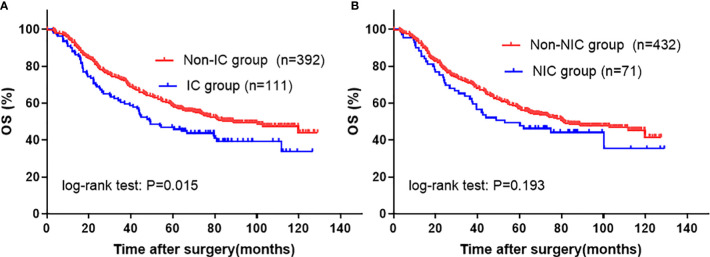
Kaplan-Meier survival analysis according to specific SC in PSM cohort. **(A)** ICs; **(B)** NICs. The significance of the difference between survival curves was calculated by the log-rank test. SC, severe complication; IC, infectious complication; NIC, non-infectious complication.

To further verify the relationship between complication and its etiology and OS, we performed a PSM analysis that helped reduce the baseline bias. As shown in **Figure 4**, after PSM, patients with SCs still showed decreased OS when compared with those without (5-year OS rate 48.5 *vs.* 59.1%; P=0.013), especially in stage III group (p=0.002). Subsequent multivariate analysis suggested that the presence of SCs was an independent prognostic factor for OS (HR, 1.529; 95% CI: 1.175–1.990; P=0.002) (**Table 4**). In addition, in the PSM cohort, univariate and multivariate analyses identified that ICs remained a significant risk factor for deteriorated overall survival (**Figure 5A** and **Table 4**). However, NICs did not show difference in long-term results compared with patients without NICs (**Figure 5B** and **Table 4**). In addition, the statistical power values of SCs, ICs, and NICs on OS were 0.975, 0.980, and 0.718, respectively, in the PSM cohort.

**Table 4:** Univariate and multivariate COX regression analysis of prognostic factors for overall survival in PSM cohort.

Variables	No. of patients	Univariate P value	Multivariate analysis*		Multivariate analysis^#^	
			HR (95% CI)	P value	HR (95% CI)	P value
Age (≥65 *vs.* <65)	199/304	0.008	1.275 (0.983–1.654)	0.067	1.278 (0.985–1.658)	0.064
Gender (Male *vs.* female)	380/123	0.289				
Preoperative albumin (≥35 *vs.* <35 g/L)	443/60	0.944				
Comorbidities (Yes *vs.* No)	169/334	0.198				
Surgery approach (Laparoscopic *vs.* Open)	61/442	0.009	0.751 (0.479–1.177)	0.212	0.748 (0.477–1.174)	0.207
Gastrectomy (Total *vs.* Partial)	152/351	0.002	1.085 (0.823–1.431)	0.563	1.080 (0.818–1.425)	0.589
Lymphadenectomy (≥D2 *vs.* <D2)	500/3	0.740				
Resection of other organs (Yes *vs.* No)	20/483	0.076	1.059 (0.582–1.928)	0.851		
Perioperative blood transfusion (Yes *vs.* No)	95/408	<0.001	1.352 (1.006–1.818)	0.045	1.384 (1.028–1.863)	0.032
Tumor size (≥5 *vs.* <5 cm)	297/206	<0.001	1.483 (1.071–2.054)	0.018	1.478 (1.069–2.044)	0.018
Macroscopic type (Bormann 3–4 *vs.* 0–2)	191/312	<0.001	1.070 (0.800–1.432)	0.648	1.062 (0.794–1.421)	0.686
Histological grade (G3/G4 *vs.* G1/G2)	333/170	0.001	1.366 (1.017–1.834)	0.038	1.360 (1.012–1.826)	0.041
Depth of invasion (T4 *vs.* T1/2/3)	247/256	<0.001	1.552 (1.161–2.073)	0.003	1.547 (1.158–2.068)	0.003
Nodal involvement (N+ *vs.* N0)	335/168	<0.001	2.378 (1.707–3.314)	<0.001	2.403 (1.724–3.349)	<0.001
Adjuvant chemotherapy (Yes *vs.* No)	238/265	0.149	0.750 (0.580–0.970)	0.028	0.754 (0.583–0.974)	0.031
**SCs (Yes *vs.* No)**	**168/335**	**0.013**	**1.529 (1.175–1.990)**	**0.002**		
**ICs (Yes *vs.* No)**	**111/392**	**0.015**			**1.567 (1.175–2.089)**	**0.002**
**NICs (Yes *vs.* No)**	**71/432**	**0.193**			**1.386 (0.978–1.963)**	**0.067**

*Multivariate analysis describing the prognosis of SCs for gastric cancer patients.

^#^Multivariate analysis describing the prognosis of ICs and NICs for gastric cancer patients.

The bold values indicate the main objects of this study.

## Discussion

Several previous studies have reported the negative impact of postoperative complications on oncological outcomes after gastric cancer resection (14–17). However, these studies failed to exclude patients who died in a short postoperative period. It is important to note that postoperative complications increase early mortality, which would overshadow the real influence of complications on long-term survival of cancer patients (33, 34). Besides, these reports (14, 17, 35, 36) did not discriminate which type of complication was the real risk factor for decreased long-term survival.

In the present study, 1,667 GC patients with curative resection were analyzed, and 10.08% of them experienced severe complications (excluding deaths within 90 days of the surgery). In the total cohort, we found that the occurrence of SCs was indeed significantly associated with shortened long-term OS, and ICs seemed to be the major cause of impaired long-term survival instead of SNICs. In addition, these findings were consistent in the PSM cohort.

The influence of complications, particularly infectious ones, on long-term survival has been described in several types of cancer (21, 22, 37). Recently, in a systematic review and meta-analysis about the effect of complications on long-term survival in GC patients with curative resection, Wang et al. identified a 40% higher risk of death in patients with complications and a much higher (86%) mortality risk in patients with infectious complications compared to those without (20); their findings match our results. Similarly, in lung cancer, an outcome reported by Andalib et al. has demonstrated that major infectious complications were the main reason for decreased rates of long-term survival and that non-infectious complications had a minor effect on this bad outcome, excluding early deaths (21).

With respect to the correlation between complications and poor survival rates, accumulated evidence (14, 38, 39) indicates that the surgical stress, especially in major surgery, induces an inflammatory response that could be worsened and prolonged by complications. It is also well established that a postoperative inflammatory response contributes to host immunosuppression by suppressing cell-mediated immunity (40, 41), especially natural killer cells and cytotoxic T lymphocytes are compromised (41), which promotes the proliferation and metastasis of residual tumor cells. Furthermore, numerous studies have confirmed that ICs have a direct effect on cancer cells’ metastatic ability by activating a bacterial antigen-mediated processes (42, 43). Indeed, in our study, the remarkable difference in overall survival rates between patients organized by the presence of complications in p-Stage III likely reflects the quantity of residual tumor cells that cause early recurrence.

Nevertheless, we must admit that complications’ relationship with decreased rates of survival is not yet clear. It is still possible that the pernicious effect of postoperative complications on long-term survival is just a confounder. Surgical technique may prove to be the reason for both occurrence of complications and decreased long-term survival. What we conclude from our study is that complications are associated with poor prognosis. Considering the curability of the complications and its potential benefit on patients’ long-term survival, it is crucial to prudently deal with complications through active intervention and remediation.

Given the fact that complications markedly compromise overall survival, to identify complication-related risk factors is therefore crucial. In the present study, older age was identified to be the only risk factor for the occurrence of complications, which was not a modifiable factor in perioperative management. In such circumstances, the prevention and early diagnosis of postoperative complications are of critical importance.

This study has some limitations. First, this is a single-center retrospective study with several confounding factors, which might bias our results and conclusions. Even though we tried our best to offset available biases with multivariate analysis and PSM analysis, some residual confounding unmeasured factors may exist. Second, there was also a lack of information about adjuvant chemotherapy. For example, the starting time of adjuvant chemotherapy among patients was unclear, which limited our further analysis of the interaction between postoperative complications and delayed adjuvant therapy on prognosis. Despite these limitations, postoperative complications are considered an important prognostic factor affecting long-term survival.

In conclusion, postoperative complications after curative resection of gastric cancer are both common and associated with poor overall survival in gastric cancer patients. And the survival disadvantage seemed to be mainly driven by infectious complications rather than non-infectious ones. However, additional multicenter prospective studies with larger sample size are required to verify this issue.

## Data Availability Statement

The raw data supporting the conclusions of this article will be made available by the authors, without undue reservation.

## Ethics Statement

The studies involving human participants were reviewed and approved by the Research Ethics Committee of West China Hospital. Written informed consent from the patients/participants’ legal guardian/next of kin was not required to participate in this study in accordance with the national legislation and the institutional requirements.

## Author Contributions

J-KH, H-YP, and L-YZ made substantial contributions to conception and design for this study. H-YP and HW collected all the data. H-YP, L-YZ, HW, X-LC, KL, and W-HZ analyzed data. H-YP, L-YZ, and X-LC drafted the article. KL, KY, X-ZC, and J-KH gave critical revision for important intellectual content. H-YP and L-YZ revised it critically for important intellectual content. KY, X-ZC, and J-KH gave final approval of the version to be published. All authors contributed to the article and approved the submitted version.

## Funding

This study was funded by (1) 1.3.5 project for disciplines of excellence, West China Hospital, Sichuan University, No. ZY2017304; (2) Post-Doctor Research Project, West China Hospital, Sichuan University (2018HXBH013).

## Conflict of Interest

The authors declare that the research was conducted in the absence of any commercial or financial relationships that could be construed as a potential conflict of interest.

## Publisher’s Note

All claims expressed in this article are solely those of the authors and do not necessarily represent those of their affiliated organizations, or those of the publisher, the editors and the reviewers. Any product that may be evaluated in this article, or claim that may be made by its manufacturer, is not guaranteed or endorsed by the publisher.

## Abbreviations

GC, gastric cancer; SC, severe complication; IC, infectious complication; NIC, non-infectious complication; CD, Clavien–Dindo; OS, overall survival; PSM, propensity score matching; HR, hazard ratio; OR, odds ratio; CI, confidence index.
