# Supplementary material for: Impact of Type of Postoperative Complications on Long-Term Survival of Gastric Cancer Patients: Results From a High-Volume Institution in China
Source: Front Oncol. 2021 Oct 11;11:587309. doi: 10.3389/fonc.2021.587309 (PMC8542852; doi:10.3389/fonc.2021.587309)
Supplement: Supplementary file 1 [file Table_1.docx]

**Table S1**. Details of postoperative complication assessed by Clavien-Dindo system.

| **All patients (n=1667)** | | | | |
| --- | --- | --- | --- | --- |
| **Type of complications** | **All complications**  **n (%)** | **CD grade I/II/III/IV** | **Non-severe complications ^a^**  **n (%)** | **Severe complications ^b^**  **n (%)** |
|  | **675 (40.49)** | **386/245/24/20** | **507(30.41)** | **168(10.08)** |
| **Infectious complications** | **536(32.15)** | **290/226/7/13** | **425**(25.49) | **111**(6.66) |
| Pulmonary infection | 306(18.36) | 122/174/1/9 | 242(14.52) | 64(3.84) |
| Intra-abdominal abscess | 33(1.98) | 7/21/3/2 | 10(0.60) | 23(1.38) |
| Wound infection | 40(2.40) | 29/11/0/0 | 34(2.04) | 6(0.36) |
| Anastomotic leakage | 5(0.30) | 0/3/0/2 | 0(0.00) | 5(0.30) |
| Pancreatic fistula | 3(0.18) | 0/2/1/0 | 0(0.00) | 3(0.18) |
| pancreatitis | 1(0.06) | 0/1/0/0 | 0(0.00) | 1(0.06) |
| Intestinal fistula | 1(0.06) | 0/0/1/0 | 0(0.00) | 1(0.06) |
| Cholecystitis | 5(0.30) | 0/5/0/0 | 1(0.06) | 4(0.24) |
| Urinary system infection | 5(0.30) | 3/2/0/0 | 5(0.30) | 0(0.00) |
| Infectious Diarrhea | 7(0.42) | 5/2/0/0 | 7(0.42) | 0(0.00) |
| Appendicitis | 2(0.12) | 0/1/1/0 | 1(0.06) | 1(0.06) |
| Bacteremia | 4(0.24) | 3/1/0/0 | 2(0.12) | 2(0.12) |
| Infection with no source | 124(7.44) | 121/3/0/0 | 123(7.38) | 1(0.06) |
| **Non-infectious complications** | **180(10.80)** | **96/60/17/7** | **109(6.54)** | **71**(4.26) |
| Gastroparesis | 51(3.06) | 21/30/0/0 | 27(1.62) | 24(1.44) |
| Intestinal obstruction | 15(0.90) | 3/11/1/0 | 7(0.42) | 8(0.48) |
| Intra-abdominal bleeding | 7(0.42) | 0/3/4/0 | 1(0.06) | 6(0.30) |
| Liver/kidney injury | 17(1.02) | 13/2/0/2 | 14(0.84) | 3(0.18) |
| Arrhythmia | 19(1.14) | 14/4/1/0 | 17(1.02) | 2(0.12) |
| Heart failure | 1(0.06) | 0/0/0/1 | 0(0.00) | 1(0.06) |
| Deep venous thrombosis | 2(0.12) | 0/2/0/0 | 0(0.00) | 2(0.12) |
| Pleural effusion | 20(1.20) | 11/5/4/0 | 14(0.84) | 6(0.36) |
| Ascites | 11(0.66) | 7/0/4/0 | 6(0.36) | 5(0.30) |
| Atelectasis | 15(0.90) | 12/2/0/1 | 13(0.78) | 2(0.12) |
| Chylous leakage | 1(0.06) | 0/1/0/0 | 0(0.00) | 1(0.06) |
| Respiratory failure | 2(0.12) | 0/0/0/2 | 0(0.00) | 2(0.12) |
| Incision fat liquefaction | 9(0.54) | 8/0/1/0 | 6(0.36) | 3(0.18) |
| Delirium tremens | 7(0.45) | 3/4/0/0 | 4(0.24) | 1(0.06) |
| Hyperthyroidism crisis | 1(0.06) | 0/0/0/1 | 0(0.00) | 1(0.06) |
| Fracture | 1(0.06) | 0/0/1/0 | 0(0.00) | 1(0.06) |
| Diaphragmatic eventeration | 1(0.06) | 0/0/1/0 | 0(0.00) | 1(0.06) |

CD, Clavien-Dindo.

^a^ Non-severe complications were defined as complications requiring a hospital stay less than 15 days with a CD grade I/II.

^b^ Severe complications were defined as CD grade III or higher complications or complications causing a hospital stay of 15 days or longer.
